# Smart Annotation of Cyclic Data Using Hierarchical Hidden Markov Models

**DOI:** 10.3390/s17102328

**Published:** 2017-10-13

**Authors:** Christine F. Martindale, Florian Hoenig, Christina Strohrmann, Bjoern M. Eskofier

**Affiliations:** 1 Machine Learning and Data Analytics Lab, Department of Computer Science, Friedrich-Alexander University Erlangen-Nürnberg (FAU), 91054 Erlangen, Germany; bjoern.eskofier@fau.de; 2 Speech Group, Department of Computer Science, Friedrich-Alexander-University Erlangen-Nürnberg (FAU), 91054 Erlangen, Germany; florian.hoenig@cs.fau.de; 3 Bosch Sensortec GmbH, Gerhard-Kindler-Strasse 9, 72770 Reutlingen, Germany; annachristina.strohrmann@bosch-sensortec.com

**Keywords:** hierarchical hidden Markov models, segmentation, smart annotation, cyclic sensor data, semi-supervised learning, annotation cost, activity recognition, gait classification, inertial sensors, wearable sensors

## Abstract

Cyclic signals are an intrinsic part of daily life, such as human motion and heart activity. The detailed analysis of them is important for clinical applications such as pathological gait analysis and for sports applications such as performance analysis. Labeled training data for algorithms that analyze these cyclic data come at a high annotation cost due to only limited annotations available under laboratory conditions or requiring manual segmentation of the data under less restricted conditions. This paper presents a smart annotation method that reduces this cost of labeling for sensor-based data, which is applicable to data collected outside of strict laboratory conditions. The method uses semi-supervised learning of sections of cyclic data with a known cycle number. A hierarchical hidden Markov model (hHMM) is used, achieving a mean absolute error of 0.041 ± 0.020 s relative to a manually-annotated reference. The resulting model was also used to simultaneously segment and classify continuous, ‘in the wild’ data, demonstrating the applicability of using hHMM, trained on limited data sections, to label a complete dataset. This technique achieved comparable results to its fully-supervised equivalent. Our semi-supervised method has the significant advantage of reduced annotation cost. Furthermore, it reduces the opportunity for human error in the labeling process normally required for training of segmentation algorithms. It also lowers the annotation cost of training a model capable of continuous monitoring of cycle characteristics such as those employed to analyze the progress of movement disorders or analysis of running technique.

## 1. Introduction

Cyclic movements intrinsic to daily life range from human motion such as running, cycling and rowing to biological signals such as the electrical activity of the heart. Understanding and quantifying these cyclic data is especially important for the analysis of movement disorders, sports, rehabilitation and general health [1,2,3]. The first step in the analysis of these cyclic data is the segmentation of them into individual cycles for direct analysis such as temporal parameter calculation (e.g., cycle time) or as training data for more complex algorithms based on pre-segmented data (e.g., stride length). Currently, there is no data processing pipeline, applicable to the movement analysis or biological signal domain, capable of unsupervised cyclic event segmentation of sensor-based data. Therefore, time-consuming manual annotation is currently required to achieve this type of segmentation, even just for validation datasets. A generic segmentation pipeline that could segment any arbitrary, previously unseen, periodic data would be valuable to these applications. The application of the pipeline would not be limited to periodic human movement data, but could be applied to any cyclic data.

Completely automatic annotation of cycles within sensor data is essentially an unsupervised segmentation task. However, the current state of the art in sensor-based systems falls mainly in the supervised domain. Segmentation algorithms can be split into three main categories of training methods: supervised, semi-supervised and unsupervised. In the supervised domain, there is an abundance of different segmentation algorithms such as dynamic time warping (DTW), longest common subsequence (LCSS) and thresholding with windowed peak detection (WPD) [4]. The main disadvantage of these supervised techniques is the annotation cost involved because each cycle in the training set must be manually segmented in order to calculate a template or threshold [5]. Reduction of this annotation cost for gait data has been addressed by using instrumented treadmills or mats [6] or by using shoes with pressure-sensitive insoles [7]. In both cases, the thresholded pressure signal is used to determine stride boundaries. However, both are restricted by the availability of such large or expensive devices, which lack in-the-field applicability. This method of automated annotation applies only to gait data.

In the semi-supervised domain, there have been several methods proposed to reduce the amount of fully-labeled data required. One of the more popular solutions is active learning or labeling [8,9]. This method identifies which sections of data would be most effective to annotate. A variation on this theme is to split the data into similar ‘populations’ and then fully label only a subsection of each population [10,11,12]. Alternatively, any shared structure between activities can be exploited [13]. These techniques have been applied only on a high level, for example to reduce the annotation cost when classifying and segmenting whole activities, however not when also segmenting these activities into individual cycles. These techniques are semi-supervised because they require labeled data, but less than for their supervised counterparts, which required fully-labeled data.

In the completely unsupervised domain, sometimes called ‘zero-resource’ because no prior information is given, there is even less literature. Within the activity recognition domain, there are some unsupervised methods, which enable segmentation of primitives within activities; however, these primitives are also found in non-cyclic data, and their boundaries vary per person, activity and method [14,15]. These methods have been demonstrated on motion capture data only on the activity level. Some online, unsupervised activity recognition methods exist [16], some of which are based on exploiting the hierarchical structure of motion data [17,18], although these do not identify primitives within each activity. Within a smart home setting, unsupervised methods have been used for classification of interleaved activities [19].

However, to the best of the authors’ knowledge, an unsupervised segmentation algorithm for both classification of activities and for segmentation of those activities into cycles has not been achieved within this domain. We focus on a method applicable to limited sensors, specifically one wearable IMU. The proposed method does not rely on a model of the human body, such that it could also be applied to other cyclic data as it relies only on the periodicity of the data. Ultimately, zero-resource techniques would completely remove the need for any level of annotation for training purposes.

This paper investigates the first steps required to achieve the aim of unsupervised segmentation for cyclic activities, without using an explicit body model. The advantage of this is that it can be used with a low number of sensors and is independent of sensor location. We present a method, based on a hierarchical hidden Markov model (hHMM), which enables the semi-supervised segmentation of uninterrupted cyclic data with a given repetition count. Due to the proposed model requiring uninterrupted cyclic data and the repetition count, it falls into the semi-supervised domain although it differs from the existing semi-supervised techniques because it requires no fully-labeled data sections.

The feasibility of this method is demonstrated using continuous walking and running data collected from a body-worn sensor. The results are compared to manual segmentation by a human. The generated model, trained in a semi-supervised manner, is then applied to simultaneous segmentation and classification of previously unseen data. The results of a fully-supervised version are used as a baseline.

## 2. Background

In order to alleviate the level of supervision required, i.e., to implement a smart annotation method, a model-based algorithm, rather than a template-based one, must be chosen. This is because a template-driven approach requires fully-labeled training data in order to form a template. However, a model-based approach can progressively learn a model by an iterative process of learning and relabeling the input data. A family of models that meet these criteria are graphical models such as hidden Markov models (HMM), conditional random fields (CRF) and semi-Markov models (SMM). Thomas et al. (2010) investigated semi-supervised SMMs for simultaneous segmentation and classification. However, their method requires some fully-labeled data, as well as prior expert knowledge of the likely sequence of events [20]. The rhythmic extended Kalman filter has also been used for unsupervised gait analysis; however, it relies on a body model, assumes walking in a straight line and requires multiple sensors firmly attached to specific locations on the subject, so although it is online and unsupervised, it requires patient-specific information and may not be extensible to other cyclic movements. Furthermore, the segmentation results were only qualitatively compared [21,22]. Markov chain Monte Carlo was also used for unsupervised activity recognition; however, individual cycles within activities were not segmented [18].

**Figure 1:**
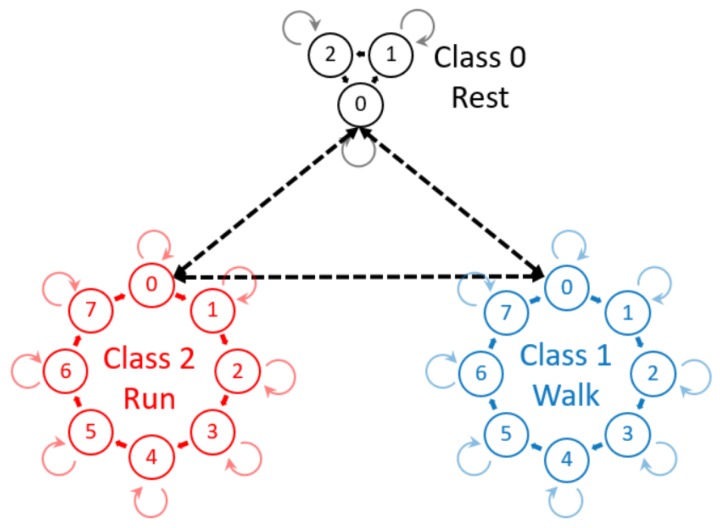
Hierarchical hidden Markov model (hHMM) consisting of three classes each of, respectively, 3, 8 and 8 internal states. The internal states are strictly circular with the possibility of recursion. The dashed arrows represent the transition model between individual HMMs that represent classes such as rest, walking and running.

HMMs are a well-understood model for sequential data, using Gaussian mixture models (GMMs) to represent the data [23,24]. Mannini et al. (2015) investigated a fully-supervised HMM for gait segmentation and Andreao et al. (2006) for ECG segmentation [6,24]. However, a four-state, circular HMM, as used by Mannini et al., does not model the transition between different classes of signal such as standing to walking [6]. Andreao et al. used expert knowledge to model the transitions within the internal structure of an ECG signal [24]. However, a more intuitive and efficient approach to address the underlying structure of cyclic data and o provide a model for the transition between different classes of a cycle is a hierarchical HMM (hHMM) [25]. The hierarchical structure allows for more efficient methods of estimating parameters and of training the individual HMMs. This hierarchical concept allows a model for each class of data and a transition model between individual HMMs (Figure 1). The above-mentioned models were all implemented in a fully-supervised manner and therefore require the entire dataset to be labeled on a per cycle basis. HMMs have been used for unsupervised, online, activity recognition and segmentation [16,17]; however, they did not further segment activities into cycles. The use of hierarchy combined with HMM has been used by both Lv et al. and Kulic et al.; however, [26] use HMMs as a weak classifier for AdaBoost, so using hierarchy, and [17] used a tree classification approach. HMMs in this field have also been used in a semi-supervised approach where the segments are initially found using velocity peaks and zero crossings, although segmentation accuracy was only analyzed for within 0.2 s [27]. This method relies on a specific shape and thresholds to perform in a semi-supervised manner.

These hHMMs have been widely used in speech recognizers [28], as well as for fully-supervised segmentation and classification of human movement data [29]. In the context of speech recognizers, the hHMM iteratively learns both the event models and the event segmentation. A similar method is applied in this paper, for application to cyclic data. However, cyclic data are made up of repetitions of the same event, whereas speech data rarely contain repeated words or phonemes, thereby reducing the ambiguity of the task. Therefore, this paper tackles their application, in a semi-supervised manner, to cyclic movement data rather than speech data. The method proposed in this paper differs from previous semi-supervised and unsupervised approaches in that it is applicable to data from a single sensor and does not inherently rely on knowledge of the location of the device, type of device or the shape of the signal. This enables its application to a single wearable device rather than specifically to motion capture data or those relying on a model of the body and at least five sensors. It also enables the influencing of the segmentation boundary based on start and end points of cycles within the training data, while also allowing these points to be arbitrary, depending on the application of the method.

## 3. Proposed Pipeline

### 3.1. Smart Annotation

**Figure 2:**
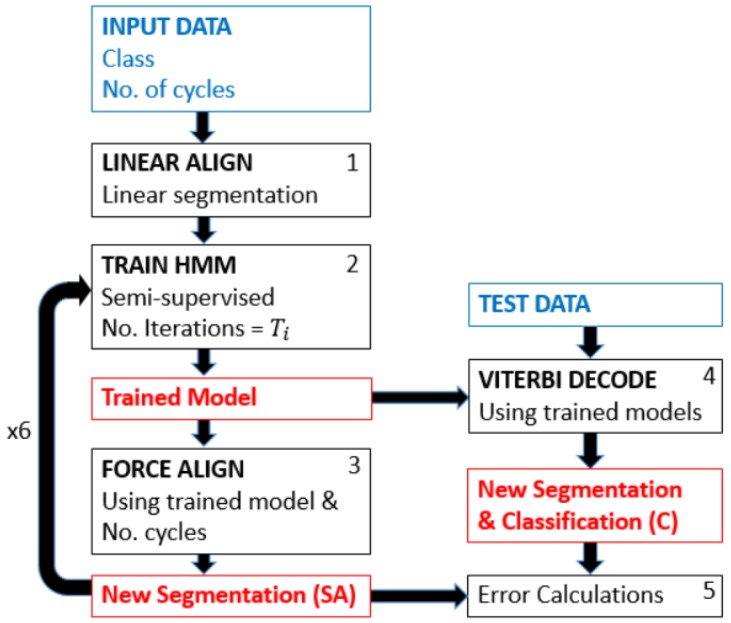
Pipeline showing the smart annotation method on the left and the use of the resulting models for simultaneous classification and segmentation of test data on the right.

The basis of this method is the concept of iterative learning from an initial estimate: the linear division of a data section by the cycle repetition number (Figure 2). As input, the pipeline requires sections of data containing one class of cycle and the number of repetitions within that section. With this information, the data are linearly divided into the given number of repetitions (Box 1, Figure 2). The resulting naive, uniform segmentation is then used for semi-supervised training of an HMM using Viterbi training [30], i.e., an efficient parameter learning alternative to Baum–Welch (Box 2, Figure 2). The training of the internal states is also initiated in a linear manner, hence making it also semi-supervised. These linear segmentations are then used to train an HMM, and the number of training iterations performed is $T_{i}$. This limit can be found empirically or set very high, if computation time is not a restriction. The resulting, trained HMM is then used to compute a forced alignment (Box 3, Figure 2). Forced alignment is the calculation of the Viterbi path to find the most probable state sequence given a known sequence of events and is commonly used and implemented within speech processing toolkits [31]. Forced alignment, in this case, simply calculates the most probable Viterbi path where the resulting path must contain the defined number of iterations. This is achieved by concatenating the given sequence, i.e., it is forced to only estimate sequences that result in the given number of repetitions. This results in new segmentation boundaries. The results of this forced alignment, the new segmentation boundaries, are then used as supervised training for the next iteration (Box 2, Figure 2). From this point on, it is a supervised model where the segmentation boundaries resulting from the forced alignment are used to split the data for training the HMM. The result of the final forced alignment is then used to calculate the segmentation error of the smart annotation model’s segmentation relative to the manually-segmented reference. The desired cycle boundary is found based on the assumption that each segment of data (primitive containing known cycle number) for training contains a known whole number of cycles, and hence, the stride boundaries will be the beginning and end of a cycle.

In order to avoid overfitting to an incorrectly segmented model (the one learned with the uniformly-segmented data), the number of training iterations, $T_{i}$, is gradually increased. For the implementation used in this paper [$T_{i}$ = 1, 2, 5, 8, 13, 21], and hence, the method looped six times between HMM training (Box 2, Figure 2) and forced alignment (Box 3, Figure 2). The number of loops was limited in this manner due to only minimal improvement with more iterations and was fixed for all experiments in this paper.

The processing of all input data segments (primitives containing known cycle number) is calculated simultaneously, preventing overfitting, as well as allowing for faster processing than running the pipeline for individual samples. This means that the models are learned simultaneously. Any number of models can be learned simultaneously; in fact, a larger variety of data to be learned simultaneously enables the model to better resolve ambiguities found when learning a model.

This pipeline, the combination of linear alignment, HMM training and forced alignment, is in this paper termed smart annotation (SA) because the resulting segmentation of the forced alignment can be used as annotations for the input data with limited supervision (repetition number and class).

### 3.2. Simultaneous Smart Annotation and Classification

A further application of this final SA model is simultaneous segmentation and classification of continuous data, with no further training than the smart annotation described. This application refers to the use of the hHMM trained using the SA method to classify and segment unseen, continuous data where no cycle numbers or segments are known. However, an additional HMM must be trained to recognize the non-movement sections that naturally occur in continuous data. This is a similar step to the simultaneous classification and segmentation carried out by [29]; however, in our implementation, the model is semi-supervised, as opposed to their fully-supervised version. If the test data are a mixture of classes, then each class present would need an HMM trained by the smart annotation method, and the transition probability between these models can be set to equal, i.e., naively initiated and not trained. With these models and the equal transition probabilities, Viterbi decoding can be used to estimate the segmentation, as well as classification of unseen test data (Box 4, Figure 2). The calculation of the Viterbi path here uses a fully-connected transition model, such as that in Figure 1. This means that no simplifications are made due to a known sequence of events, and the model has less prior knowledge than in the previous case, i.e., neither the number of events, nor the sequence.

## 4. Dataset

**Figure 3:**
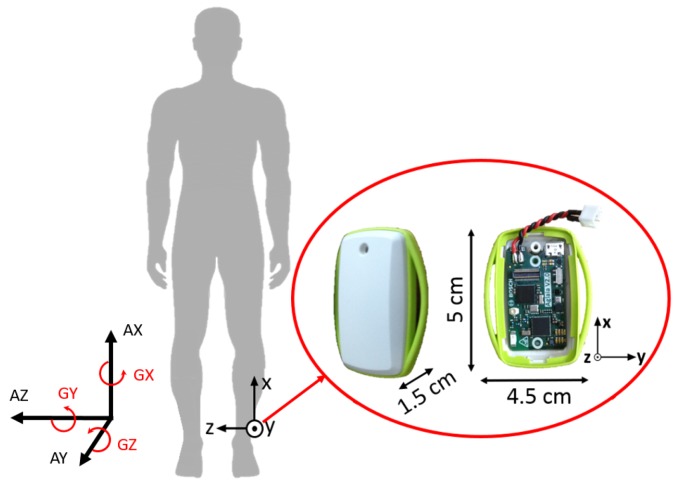
Sensor placement, size and orientation. Three-dimensional acceleration (AX, AY, AZ) and three-dimensional gyroscope (GX, GY, GZ).

**Figure 4:**
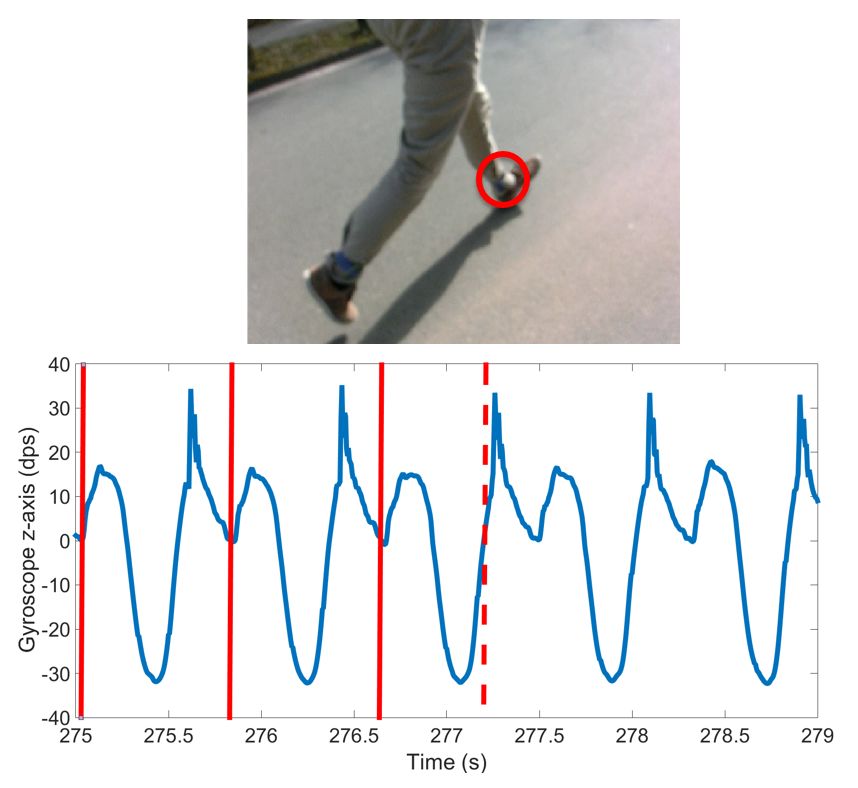
Image of manual labeling tool showing that the signal is the gyroscope sagittal plane (GZ, blue line) of the left foot. Solid vertical lines represent the already annotated cycles. The dashed vertical line corresponds to the shown camera frame. The circle in video frame shows the sensor location.

In order to investigate the effectiveness of this smart annotation method, a dataset of cyclic activities, in this case walking and running, was collected using an inertial measurement unit (IMU) within the Bosch development platform, which recorded acceleration (±8 g) and the rate of change of the angle (2000 dps) with a frequency of 200 Hz (dataset available at www.mad.tf.fau.de/research/activitynet). Eighteen participants, 14 male and 4 female, with an average age of 28.3 ± 3.0 years and an average height of 177.1 ± 8.3 cm, performed a sequence of standing, walking and running while wearing the above-mentioned sensor above their left ankle (Figure 3). This paper aims to provide a method applicable to any cyclic data, including where just one sensor is available. The data were collected ‘in the wild’ in that it was an outdoor setting with varying surfaces, with a prescribed circuit, but self-selected speeds, and the transition points were landmarks. The data collection was continuous per person. This was simultaneously video recorded (30 Hz) for annotation purposes. Reference labels were found by simultaneous analysis of video and sensor signal as illustrated in Figure 4. All subjects gave written informed consent, prior to the data collection, in accordance with the ethical committee of the medical faculty at the university.

**Figure 5:**
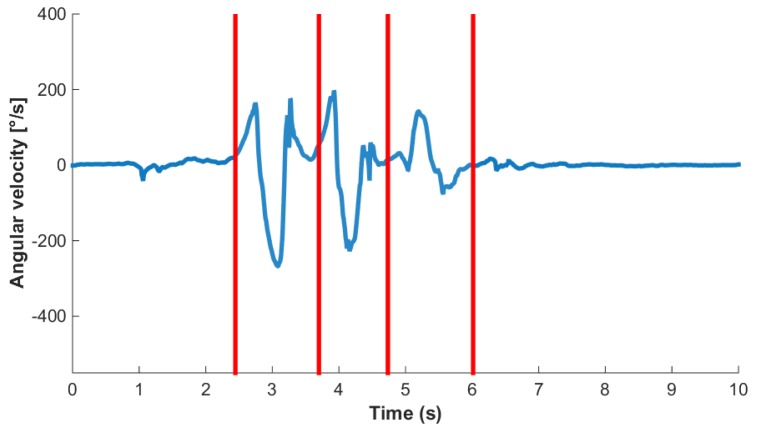
Sample gyroscope signal (blue line) consisting of three strides bounded by rest. Segment boundaries (SB) are shown as vertical lines.

A cycle, in this case a stride, was labeled such that the beginning of the first stride and end of the last stride in a sequence are data points corresponding to the sensor being stationary. In the case of standing this means that the sensor was vertically above the ankle. Therefore, each stride border (SB) within a bout of walking or running was labeled as the point where the sensor was vertically above the ankle. An example sensor signal with SB labels and classes is shown in Figure 5. This definition enables the precise detection and definition of the beginning of an activity, i.e., the initial movement of the sensor. It also enables the labeling and counting of a low number of stride events. For example, if a subject takes one stride, from standing on the left foot, then there will only be one heel strike of the left foot. If a stride were defined as heel strike to heel strike, then this single stride would not be segmented or classified as a stride. Segmenting strides using the mid-stance or stationary section of a stride has two main benefits: it can immediately be used in algorithms that rely on identification of the zero velocity phase, and it allows for easy isolation of sections of whole cycles as one may just find standing phases and then count the cycles between.

Human annotators, familiar with data from human movement both as IMU data and as camera data, manually segmented all strides and labeled each stride as walking or running. Each labeler annotated an independent section of the data. All instances of standing were labeled as rest. Any section of data that was not within these three categories was labeled as transition and ignored for evaluation purposes. Each labeler annotated an independent section of the data. The IMU data and video were synchronized using a repetitive movement of the sensor at the beginning and end of each recording. A total of 2263 walking strides and 1391 running strides were manually labeled with a minimum of two separate examples of rest (standing) per person.

## 5. Implementation Details

### 5.1. Smart Annotation

The processing pipeline (Figure 2) was implemented using the Java Speech Toolkit (JSTK) [32]. The number of internal states per HMM was chosen via a grid search (Table 1). The values for the grid search were chosen partially empirically and partially based on the literature [5,23,33]. The range of internal states used was chosen on the assumption that these numbers of internal states are meaningful for gait, in that gait is often divided into four or eight phases [33]. Ten states were also included to investigate if a higher number of states than is physically meaningful might also be useful. The number of centers for the initialization of the Gaussian mixture models (GMM) used to model the data was trivially initialized to 10. The densities of these were calculated using 10 estimation maximization (EM) iterations. A diagonal covariance matrix was used.

### 5.2. Simultaneous Smart Annotation and Classification

A further application of this hHMM is simultaneous segmentation and classification of continuous, ‘in the wild’ data, with no further training than the smart annotation described. The number of different classes of cycles was two in this case: walking and running. For this part of the pipeline, an HMM representing rest or standing was also trained, due to the test data including sections of rest. The number of internal states for rest was fixed to three. In order to estimate the probable sequence of events of a test signal, i.e., calculate the Viterbi path for a signal, the probability of occurrence of each class is also needed. In order to keep the solution general, all possible events were given an equal probability of occurring, and only the current event was taken into account.

For comparison, an hHMM supervised with the SB, but not the internal states of each stride, was trained and applied to simultaneous segmentation and classification of the same test data.

### 5.3. Data Preparation

A variety of combinations of axes was chosen to compute features with, and the best was selected via a grid search (Table 1). The gyroscope sagittal plane data featured in all combinations due to its common use in mobile gait analysis [5]. The features chosen were variance, the first three coefficients of the second order polynomial fit, as well as the raw data. The features were calculated from the unfiltered, but calibrated data. These were calculated using a sliding window approach where the overlap was one sample less than the window size. These window sizes were chosen up to the average cycle length (Table 1). A simple feature set was chosen to illustrate the general applicability of this method, as well as to reduce processing time. The coefficients of the polynomial fit were chosen as they describe the shape of the data within a relatively large window with minimal features (e.g., a window of 100 samples needs only three values to describe its shape). All features were normalized, per person, to remove inter person differences in amplitude and mean. It is assumed that this normalization, for application purposes, could be calculated from just a short running trial or estimated based on the height and weight of the user. It also allows the method to potentially be used based on the cyclic nature of the data and not on the placement of the sensor.

### 5.4. Evaluation Metrics

#### 5.4.1. Smart Annotation Pipeline

In order to assess the segmentation accuracy of the presented pipeline, the mean absolute error in segmentation of the SB was calculated. This is the difference between each estimated SB location and that found by the human annotators. The mean absolute error, on a per cycle basis, of each cycle duration (stride time) was also calculated.

#### 5.4.2. Simultaneous Smart Annotation and Classification

For the evaluation of the effectiveness of classification, the F1-score, sensitivity and specificity were calculated, as well as the confusion matrix. The segmentation evaluation metrics mentioned above are also reported. In this case, each estimated cycle is matched to the corresponding ground truth cycle by finding the two cycles with the maximum overlap. If two steps are estimated where in the ground truth there is only one, then the estimated step with the greatest overlap is used for segmentation and cycle duration error calculation. If an estimated step is two steps in the ground truth, then again, the ground truth step with maximum overlap to the estimated step is used for error calculation. This erroneous segmentation boundary will be reflected in a very high cycle duration error due to the fact that all cycles are contiguous. This allows the segmentation and cycle duration errors to reflect the accuracy in segmentation when a step is correctly identified. Any false positives or false negatives will be reflected by the classification accuracy results. The error and classification accuracies are calculated for each test person and then averaged per class, on a per sample basis. Samples where the human annotator was unsure of the class, as described previously, were disregarded.

#### 5.4.3. Train and Test Sets

The data from 12 randomly chosen subjects (training set) were used for training both the smart annotation and the simultaneous segmentation and classification models. The data were split randomly into sequences of 5–20 consecutive cycles. The same sequences were used for supervised and smart annotation training purposes, where for supervised training, the SB were given, and for smart annotation, only the repetition number was given. The longer the sequence that the HMM has to learn, the less likely it is to learn a good model. Therefore, the range of 5–20 cycles was chosen as a compromise between lowering annotation cost and keeping the sequence short enough for the HMM to be able to resolve ambiguities in the model.

The remaining six subjects (test set) were used for testing and comparison of the models. This test data were not split, but remained a continuous section of data containing rest, walking and running.

A model was trained for all combinations of the parameter choices in Table 1. In the case of smart annotation, the model that achieved the lowest segmentation error was used for the calculation of evaluation metrics. This model was then also applied to the simultaneous segmentation and classification task. This is the best model choice when segmentation of the input sequences is the only outcome. However, this may not be the optimal model when inter-class transitions are included.

In the case of supervised simultaneous segmentation and classification, the best model was chosen via a three-fold cross-validation of the training set, using the highest F1-score as the criteria for the best model. No subject was both in the training and test set within the cross-validation folds. Using these model parameters from the best model, the whole of the training set (training set) was then used to train a new model. The resulting model was then applied to the test set. For fair comparison of the effect of using smart annotation as opposed to supervised input to the model, the smart annotation model with identical parameters to the best supervised model was also applied to the test set and evaluated.

The same train-test split (12:6) was used for the supervised and semi-supervised methods, and parameter choice was performed via three-fold cross-validation of the training set, ensuring that no data from a subject used for training was used for testing, showing the generalizability of the model.

## 6. Results

### 6.1. Smart Annotation Pipeline

The best model for smart annotation was achieved using a window size of 0.5 s, the sagittal plane rate of change of angle (GZ) and four internal states per class. We will refer to this model as SA-GZ4 (Table 2). The mean absolute error of the segmentation of SB, relative to the manually segmented reference, was 0.041 ± 0.020 s, and the mean absolute cycle duration (stride time) was 0.032 ± 0.005 s (Table 2).

The best model according to the fully-supervised hHMM (see the following section) was applied to smart annotation alone (SA-AXGZ8, Table 2). The results are slightly worse, achieving a segmentation error of 0.054 ± 0.010 s, and the mean cycle duration error was 0.035 s, almost identical to that of the SA-GZ4 model, however with a worse standard deviation of 0.055 s.

While small differences were found using the grid search, the overall results did not vary drastically within the grid search with the mean segmentation error being 0.055 ± 0.010 s and the mean stride duration error being 0.040 ± 0.005 s. This implies that the parameter choice, while helpful, is also fairly robust to non-optimal selection.

### 6.2. Simultaneous Smart Annotation and Classification

#### 6.2.1. Classification and Confusion

The model found to be the best for the smart annotation (SA-GZ4) was then applied to the task of simultaneous segmentation and classification achieving an overall F1-score of 93.2% (Table 3). The F1-score for rest was naturally high, due to its simplicity; however, the F1-scores for walking and running were relatively low at roughly 90% each. The confusion matrix shows some misclassification between walking and running (Table 4).

To give a baseline for comparison, a supervised model was used to segment and classify the test data. The best model choice for this was the gyroscope sagittal plane (GZ) and the vertical acceleration plane (AX), with a window size of 0.5 s and eight internal states (Sup-AXGZ8). This model achieved an overall F1-score of 96.8% (Table 5) and similar misclassifications between walking and running (Table 6).

**Figure 6:**
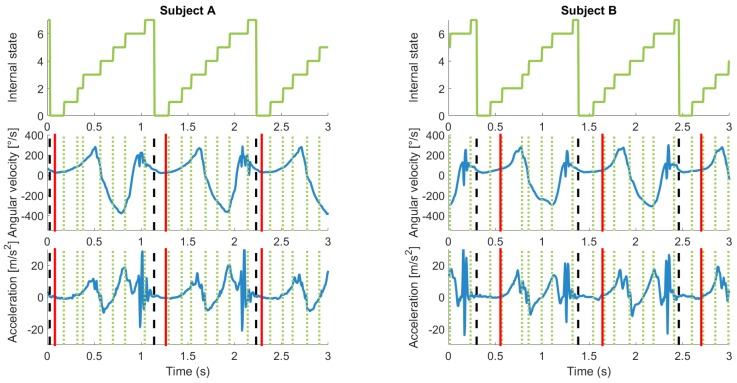
Example of estimated and ground truth labels for two subjects. Left column, Subject A; right column, Subject B. **Top row**, internal states of hHMM with eight internal states. **Middle row**, gyroscope sagittal plane signal. **Bottom row**, vertical acceleration plane. The vertical dotted lines correspond to the internal states. The dashed vertical lines correspond to the estimated start of each cycle. The vertical solid lines refer to the reference labels. The reference labels are inconsistent: for Subject A, they appear at a local minimum and for Subject B not at the local minimum. Some internal states seem to correspond to meaningful data points such as minimums and maximums.

The best model choice according to the supervised model was then also applied when the initial model was trained in the smart annotation method (SA-AXGZ8). The classification and confusion results are shown in Table 7 and Table 8. The results are very similar to the fully-supervised version, sometimes even achieving better results such as for the walking F1-score. An example of the segmentation results on an internal state level can be seen in Figure 6.

#### 6.2.2. Segmentation Error

The segmentation errors for the above-mentioned models show that the segmentation achieved for the AXGZ8 model is comparable for the supervised and smart annotation approaches, while the segmentation error achieved for the model chosen via the best segmentation error for the training set (SA-GZ4) achieved considerably higher errors (Table 2).

## 7. Discussion

The results for the smart annotation pipeline show that given a known number of iterations, cyclic data can be segmented, with an absolute segmentation error of 0.041 ± 0.020 s, which is on average 4.0% of an individual cycle duration. This is achieved with a lower annotation cost than a fully-supervised version by the order of about 10. The smart annotation alone errors were overall lower than those resulting from the segmentation and classification of test data, using the model trained by smart annotation. This is possibly due to the the fact that the smart annotation alone results necessarily contained the correct number of steps due to the forced alignment stage. This need for the repetition number is a limitation of this method. However, obtaining the number of cycles in a given section of data is still a lower annotation cost than the full, manual segmentation of the same data section needed for supervised training. Furthermore, the test data were a continuous sequence with mixed classes and transitions between these classes; the training set was small; the test subjects were not included in training; finally, the results were based on the model trained by the smart annotation method not a fully-supervised method.

The classification results and confusion matrix for the smart annotation technique and the model trained in a fully-supervised manner are comparable. The F1-scores of the method using smart annotation and the equivalent fully-supervised version differ by less than 1.0% (absolute). This implies that the smart annotation does not greatly change the accuracy of the model, while it does noticeably change the annotation cost. This also shows that a model can be trained on semi-supervised training data, a subset of the available data, and still be applicable for the annotation of the complete dataset.

The classification results of the model trained with the smart annotation method are not only comparable to the supervised version of the algorithm implemented in this paper, but also correspond to the values found by Panahandeh et al., who used a fully-supervised simultaneous segmentation and classification method [29]. The results are comparable because the datasets are of a similar size and age range, although Panahandeh et al. used a training set of 10 people and a test set of four people. Furthermore, the models and features are similar. Panahandeh et al. found true positive values for rest, walking and running of 99.0%, 97.5% and 98.5%, respectively [29]. The smart annotation method, requiring a much lower annotation cost, achieved true positive values for rest, walking and running of 99.5%, 97.8% and 99.6%, respectively.

Human error in the reference label would have influenced the error calculations, in the form of inconsistent labeling. An example of this is shown in Figure 6 where the choice of SB is closer to the beginning of the mid-stance phase for Subject A and more towards the end for Subject B. Although this is shown as an error between the result and reference, the resulting segmentation is not unreasonable; in fact, the outcome of the algorithm may be more consistent than the human labelers. The errors were also influenced by the model allowing internal state sequences where some internal states last only one sample and correspondingly have a low probability, as estimated by the model. From our physical understanding of walking, we could force the model to not consider possibilities where the internal states are too short. However, this may limit the model’s general applicability; therefore, we did not correct for this in this study. This also shows that the method is robust to noisy annotations.

As illustrated in Figure 6, the internal states could be further investigated. Once calibrated with a gold standard such as that used by [2], they could be used to segment cycles, as well as phases. There is no reliable reference for the gait phases for this dataset; however, from visual inspection, it can be seen that the changes of state often correspond to local maximums or minimums. The most notable is State Change 6–7, which corresponds closely to the heel strike peak according to Rampp et al. [34]. In domain-specific applications, important phases within a cycle could be identified using the internal states.

The differentiation of the classes of walking and running could still be improved using a post-processing method such as what Mannini et al. propose [35]. A possible reason for some of the misclassifications between these two classes is both their similarity and the subjective view of the annotators as to the differentiation of the two classes. This is due to the fact that the dataset contained transitions between running and walking that were not instantaneous. Therefore, misclassifications of less than 1% are to be expected.

One major advantage of this method is that it does not intrinsically rely on a model of the human body, meaning that it could potentially be applied to any cyclic data. It does not rely on a specific shape of a cycle or a specific point of interest at the boundaries; rather, it relies on the data being cyclic. This means that it could potentially be applied to a variety of sensor modalities and positions.

## 8. Conclusions

The analyses of cyclic movements are restricted by a high annotation cost due to the currently available fully-supervised algorithms for segmentation on a per cycle basis. Several semi-supervised techniques exist that have a lower annotation cost; however, they still require full, manual segmentation of a subsection of the dataset. The available unsupervised methods either require multiple sensors and a model of the body or predictably segment only on the activity level. This paper introduces and demonstrates a method for smart annotation of cyclic data from just one sensor, using an hHMM. Our method had a mean absolute segmentation error of 0.041 ± 0.020 s and a mean cycle duration error of 0.032 ± 0.005 s. When applied to simultaneous segmentation and classification, our method had a similar classification accuracy, 96.2%, to that of the fully-supervised version of the same model, 96.8%, but achieved this with a much lower annotation cost by a factor of about 10. This is a first step towards zero-resource learning of the cyclic data intrinsic to daily life. The application of this method is not limited to cyclic movement data, but could also be applied to other cyclic data in the wearable computing domain, such as other biological signals, as it is not restricted by assumptions based on sensor type or location. This method lowers the annotation cost of training a model capable of continuous monitoring of cycle characteristics such as the progress of a movement disorder or analysis of running technique.

## 9. Outlook

The presented method still requires the number of cycles per data sample and the class for training; however, these labeling costs are still lower than that of a fully-labeled training set required for state of the art segmentation methods. This need for cycle number and class are the next problems to be tackled, along with automatically splitting the data into the small sections required to train an hHMM. Based on the methods presented in this paper, we will now investigate the automatic detection of the number of cycles present in a section of cyclic data. A clustering method could be investigated for the solving of the class problem for the input data.

The method is based on the concept that hHMM can cope with noisy labels and therefore could also be applied simply in the case of noisy data or a mixed case of some noisy data and some data labeled by cyclic sections. A further application of this model by exploiting the nature of an HMM makes use of the internal states. As seen in Figure 6, the internal states may correspond with a meaningful physical state. Finally, we would like to continue testing our method on cyclic sensor signals common to the wearables field and investigate the effect of dataset size on the accuracy of the smart annotation.

## Figures and Tables

**Table 1:** Rough grid search for hHMM parameter optimization.

Type of Parameter	Parameter Value
Number of states per HMM	4, 8, 10
Sliding window length (s)	0.25, 0.50, 1.00
Axes combinations (See Figure 3)	AXGZ, GYGZ, AXAYAZGXGYGZ, AYGZ, GZ

**Table 2:** Mean absolute error in segmentation of stride borders (SB) relative to manual segmentation and mean absolute error in cycle duration (stride time).

Method	Model	Segmentation Error	Cycle Duration Error
Smart annotation alone	SA-GZ4	0.041 ± 0.020 s	0.032 ± 0.005 s
Smart annotation alone	SA-AXGZ8	0.054 ± 0.010 s	0.035 ± 0.055 s
Segmentation & classification, using SA	SA-GZ4	0.101 ± 0.069 s	0.098 ± 0.088 s
Segmentation & classification, using SA	SA-AXGZ8	0.063 ± 0.029 s	0.056 ± 0.025 s
Supervised segmentation & classification	Sup-AXGZ8	0.069 ± 0.029 s	0.064 ± 0.014 s

**Table 3:** Classification results, using the smart annotation model (SA-GZ4), on continuous test data on a per sample basis. All calculated per person and then per class and displayed as a percentage.

	Overall	Rest	Walking	Running
Sensitivity	91.8	99.4	86.6	89.5
Specificity	98.8	99.2	99.2	97.9
F1-score	93.2	98.5	90.7	90.5

**Table 4:** Confusion matrix, using the smart annotation model (SA-GZ4), of continuous data including walking, running and rest on a per sample basis. All calculated per person and then per class and displayed as a percentage.

	Classified Rest	Classified Walking	Classified Running
True Rest	99.8	0.2	0.0
True Walking	1.1	96.7	2.0
True Running	0.0	1.5	98.5

**Table 5:** Classification results, using the supervised model (Sup-AXGZ8) on continuous test data on a per sample basis. All calculated per person and then per class and displayed as a percentage.

	Overall	Rest	Walking	Running
Sensitivity	96.6	98.7	93.1	97.9
Specificity	99.1	99.7	98.8	98.7
F1-score	96.8	98.8	95.0	96.5

**Table 6:** Confusion matrix, using the supervised model (Sup-AXGZ8), of continuous data including walking, running and rest on a per sample basis. All calculated per person and then per class and displayed as a percentage.

	Classified Rest	Classified Walking	Classified Running
True Rest	99.1	0.9	0.0
True Walking	0.5	98.1	1.4
True Running	0.0	1.1	98.9

**Table 7:** Classification results, using the smart annotation model (SA-AXGZ8), on the continuous test data on a per sample basis. All calculated per person and then per class and displayed as a percentage.

	Overall	Rest	Walking	Running
Sensitivity	95.6	98.7	91.3	96.9
Specificity	99.2	99.5	99.4	98.6
F1-score	96.2	98.6	94.3	95.8

**Table 8:** Confusion matrix, using the smart annotation model (SA-AXGZ8), of continuous data including walking, running and rest on a per sample basis. All calculated per person and then per class and displayed as a percentage.

	Classified Rest	Classified Walking	Classified Running
True Rest	99.5	0.5	0.0
True Walking	0.7	97.8	1.5
True Running	0.0	0.4	99.6

## Abbreviations

The following abbreviations are used in this manuscript:

hHMM	hierarchical hidden Markov models
SA	smart annotation
IMU	inertial measurement unit
SB	stride border
GMM	Gaussian mixture model
